# Targeted delivery of an ADP-ribosylating bacterial toxin into cancer cells

**DOI:** 10.1038/srep41252

**Published:** 2017-01-27

**Authors:** N.-I. Zahaf, A. E. Lang, L. Kaiser, C. D. Fichter, S. Lassmann, A. McCluskey, A. Augspach, K. Aktories, G. Schmidt

**Affiliations:** 1 Institute for Experimental and Clinical Pharmacology and Toxicology, University of Freiburg, Albertstr. 25, 79104 Freiburg, Germany; 2 Institute of Surgical Pathology, Medical Center - University of Freiburg, Breisacherstr. 115a, 79106 Freiburg, Germany; 3 Faculty of Medicine, University of Freiburg, Freiburg, Germany; 4 Comprehensive Cancer Center, Freiburg, Germany; 5 BIOSS Centre for Biological Signalling Studies, University of Freiburg, Freiberg, Germany; 6 German Consortium for Translational Cancer Research (DKTK), Freiberg, Germany; 7 Department of Microbiology and Immunobiology, Harvard Medical School, 77 Avenue Louis Pasteur, Boston, MA02115, USA

## Abstract

The actin cytoskeleton is an attractive target for bacterial toxins. The ADP-ribosyltransferase TccC3 from the insect bacterial pathogen *Photorhabdus luminescence* modifies actin to force its aggregation. We intended to transport the catalytic part of this toxin preferentially into cancer cells using a toxin transporter (Protective antigen, PA) which was redirected to Epidermal Growth Factor Receptors (EGFR) or to human EGF receptors 2 (HER2), which are overexpressed in several cancer cells. Protective antigen of anthrax toxin forms a pore through which the two catalytic parts (lethal factor and edema factor) or other proteins can be transported into mammalian cells. Here, we used PA as a double mutant (N682A, D683A; mPA) which cannot bind to the two natural anthrax receptors. Each mutated monomer is fused either to EGF or to an affibody directed against the human EGF receptor 2 (HER2). We established a cellular model system composed of two cell lines representing HER2 overexpressing esophageal adenocarcinomas (EACs) and EGFR overexpressing esophageal squamous cell carcinomas (ESCCs). We studied the specificity and efficiency of the re-directed anthrax pore for transport of TccC3 toxin and established *Photorhabdus luminescence* TccC3 as a toxin suitable for the development of a targeted toxin selectively killing cancer cells.

Pharmacological treatment of tumor cells is mainly based on causing damage of dividing cancerous and non-cancerous cells. This is accompanied by severe side effects. Therefore, more specific drugs have been developed. Among them are kinase inhibitors and antibodies. Unfortunately, no surface molecules exclusively expressed on tumor cells have been uncovered, which could be used to selectively attack transformed cells. However, much effort has been spent on the generation of specific antibodies against overexpressed surface proteins. Approaches have included development of immunotoxins composed of an antibody directed against the overexpressed cell surface receptor and a catalytic domain of a toxin fused to it^1^,^2^,^3^. For higher selectivity of this artificial fusion-protein the toxin is devoid of its natural binding component^4^. Following the interaction of the chimeric protein with surface protein, the immunotoxin is endocytosed and has to be released from the endosome into the cytosol. This is an endogenous property of some toxins, which are able to cross the endosomal membrane. For this purpose acidification of the endosome and proteolytic cleavage of the toxins catalytic part is necessary^5^,^6^,^7^. Besides antibodies, growth factors have also been used as receptor binding component of bacterial toxins (named targeted toxins), because growth factor receptors are frequently overexpressed on tumor cells. Well established immunotoxins and targeted toxins contain the ADP-ribosyltransferases *Pseudomonas* exotoxin (PE) or diphtheria toxin (DT)^3^,^8^,^9^. Both modify elongation factor (EF) 2, leading to the inhibition of protein synthesis, cell cycle arrest and cell death^10^. Truncated diphtheria toxin covalently attached to interkeukin-2 (Denileukin diftitox, ONTAK) is approved for the treatment of cutaneous T-cell lymphomas (CTCL). It binds selectively to the interleukin-2 receptor (CD25+) on lymphocytes and is taken up by endocytosis^11^. Moreover, ONTAK is effective in the maintenance therapy for peripheral T-cell lymphomas with low appearance of adverse effects^12^. Therefore, other well characterized bacterial toxins encompassing high catalytic activity and specificity against target molecules should be tested as possible therapeutic strategies against cancer.

Some bacterial toxins are composed of two separate proteins: a pore-forming transport unit and a catalytic part delivered through the pore into the cytosol of mammalian cells^13^,^14^,^15^,^16^. Anthrax toxin consists of protective antigen (PA) and two enzymes: lethal factor (LF) and edema factor (EF)^17^. Following proteolytic activation, PA assembles to a heptameric pore initiated by receptor binding, endocytosis and acidification^18^,^19^. It inserts into the endosomal membrane to transport the effectors LF and EF into mammalian cells^20^. In artificial systems such a pore can be used to transport a variety of bacterial toxins into mammalian cells either following endocytosis or directly through the plasma membrane by acidification of the extracellular milieu^21^,^22^.

In our study, we used PA as a double mutant (N682A, D683A) which cannot bind to the two natural anthrax receptors, tumor endothelium marker-8 (TEM8) and capillary morphogenesis protein 2 (CMG2)^15^,^23^. For selective binding to tumor cells each mutated monomer (mPA) is fused either to EGF or to an affibody directed against the human EGF receptor 2 (HER2). Both fusion toxins have been shown to bind their cellular receptors and to mediate entry of different proteins into cells^24^.

Several protein toxins can be transported through the re-directed anthrax heptamer by fusing them to the N-terminal PA-binding domain of the natural effector LF (LFN). The truncated LFN has no catalytic activity. Here, we used the ADP-ribosyltransferase TccC3 (aa 679–960, hypervariable region - C3hvr) of the insect pathogen *Photorhabdus luminescence* as fusion partner. This toxin modifies actin at threonine-148, inhibits its interaction with actin-binding proteins like thymosin-β4 and forces actin aggregation^25^,^26^. Therefore, actin-dependent processes cannot be executed anymore. The objective of these studies was to evaluate a cellular model system for testing specificity and efficiency of several bacterial toxins to be transported by the re-directed anthrax pore. Moreover, we intended to study suitability of the *Photorhabdus luminescence* toxin TccC3 catalytic part to be transported by mPA for killing cancer cells. We chose two cell lines of the two main histotypes of esophageal cancer, esophageal adenocarcinomas (EACs) and esophageal squamous cell carcinomas (ESCCs), which are characterized by divergent overexpression of the two targeted receptors, EGF-receptor (EGFR) and HER2. OE21 cells as a member of the ESCC cell lines show high expression of EGFR forming mainly EGFR homodimers and only some EGFR/HER2 heterodimers, whereas OE33 cells as representative of EACs exhibit strong HER2 expression with the formation of HER2 homodimers and a lower amount of EGFR/HER2 heterodimers^27^.

## Results

We have shown previously that the *Photorhabdus luminescence* toxin TccC3 catalyzes the ADP-ribosylation of actin at threonine-148. This modification blocks the interaction of actin with thymosin-β4, an actin sequestering protein resulting in uncontrolled actin polymerization and subsequent cell death.

### Generation of a TccC3 fusion protein which can be transported through the PA pore

**Figure 1:**
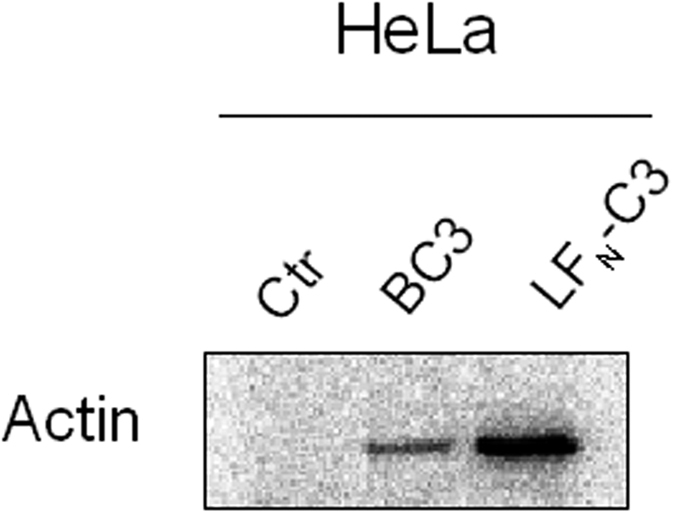
Recombinant LFN-C3 is active: HeLa cell lysate was incubated with the purified recombinant fusion protein LFN-C3 in the presence of radio-labeled NAD^+^. As negative control lysate was incubated with radio-labeled NAD^+^ without LFN-C3, as positive control with NAD^+^ plus *Photorhabdus* complete TccC3 ADP-ribosyltransferase (BC3). Shown is the readout of the dried SDS-PAGE as a typical result of three independent experiments.

For transport through the re-directed PA-pore we generated a fusion toxin composed of the N-terminal 263 amino acids of anthrax lethal factor (LFN) and the hypervariable region of TccC3 (aa 679–960, C3hvr)^28^,^29^. LFN has no catalytic activity. It binds the PA heptamer and allows the transport of proteins through the PA pore. C3hvr is the minimal catalytically active domain of the *Photorhabdus* toxin TccC3 30. First, we tested whether the recombinant fusion toxin (named LFN-C3) was catalytically active. Therefore, HeLa cell lysates were incubated with LFN-C3, the full *Photorhabdus* TccC3 toxin as a positive control and without the toxin as negative control in the presence of ^32^P-radio-labeled NAD^+^. The active catalytic domain of TccC3 catalyzes ADP-ribosylation of actin and therefore, actin is radio-labeled. This can be visualized following separation of the proteins by SDS-PAGE and exposure of the dried gel to a phosphor imager plate (Fig. 1). The data indicate that the fusion protein is folded correctly and is able to modify actin in cell lysates.

**Figure 2:**
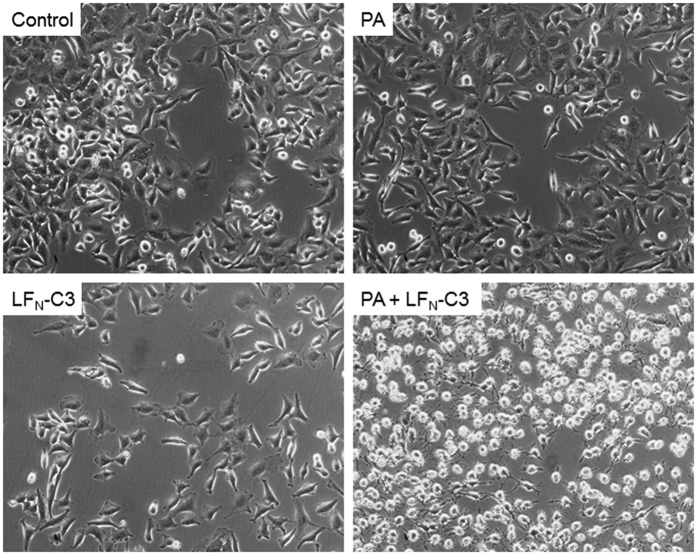
Cell rounding induced by LFN-C3: HeLa cells were treated with PA (10 nM), LFN-C3 (8 nM), a combination of both or left untreated as indicated at 37 °C. Photographs were taken 2 h following intoxication. Shown is a typical result of 3 independent experiments.

In a second experiment, the activity of the fusion toxin together with the anthrax PA (wildtype) was tested on cells. HeLa cells were treated with PA or LFN-C3 alone, with PA and LFN-C3 together or left untreated as indicated in the figure. Following 2 h of incubation photographs of the cells were taken. As shown in Fig. 2, only cells treated with PA together with the fusion toxin rounded up, showing that the fusion toxin is transported through the PA pore and is active in the cells. In contrast, the pore-forming PA alone nor LFN-C3 alone does change cellular morphology. To further exclude purification artifacts, we generated catalytically inactive LFN-C3(E943A) and a PA-mutant which can form oligomers on the cell surface without generating a functional pore (PA(F427A), Φ-clamp^31^). We combined each mutated protein with the respective wildtype counterpart for intoxication experiments with OE21 cells. As expected, only PA wildtype combined with LFN-C3 wildtype induced cell rounding (Fig. S1).

### Specificity of the re-directed toxin transporter

**Figure 3:**
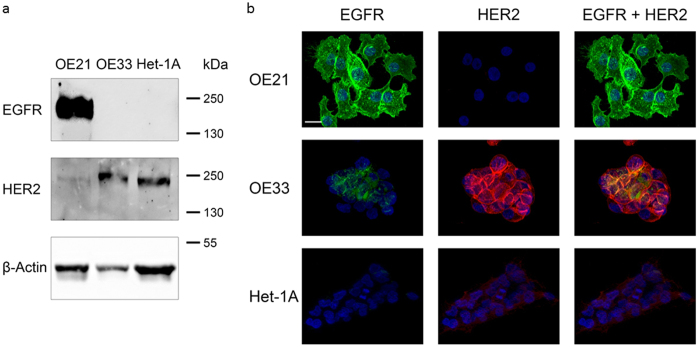
OE21 cells express high amounts of EGF receptors and little HER2, whereas OE33 cells express HER2 and a small amount of EGFR. (**a**) Western blot as a typical result of more than three independent experiments (**b**) immunofluorescence with specific antibodies against EGFR and HER2 as indicated, shown are typical stainings of more than 50 cells monitored.

**Figure 4:**
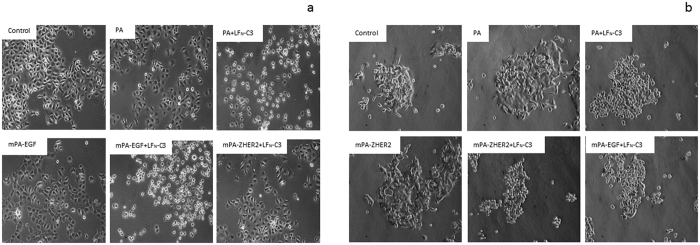
Cell specific cell rounding induced by LFN-C3 mediated by different PA transporters: OE21 (**a**) and OE33 (**b**) cells were incubated with the following proteins as indicated: PA (10 nM), mPA-EGF (10 nM), mPA-ZHER2 (10 nM), LFN-C3 (8 nM). Photos were taken after 4 h of incubation. A typical result of 3 independent experiments is shown.

**Figure 5:**
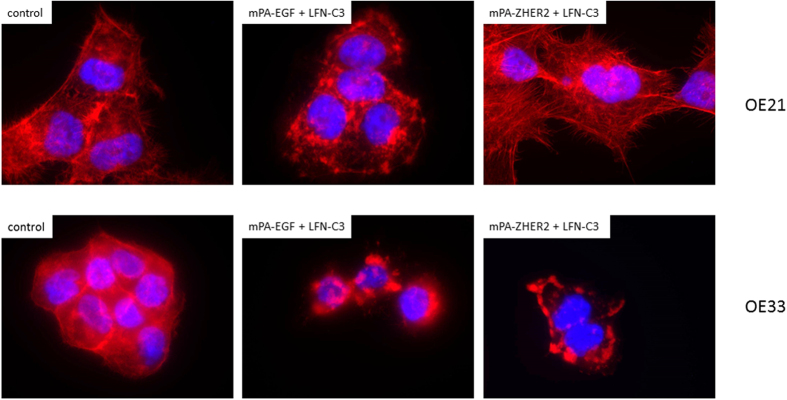
Uncontrolled actin clustering in OE cells induced by LFN-C3 mediated by different PA transporters: OE21 cells and OE33 cells were treated with mPA-EGF (10 nM), mPA-ZHER2 (10 nM), LFN-C3 (8 nM) as indicated. After 90 min cells were fixed and stained with Rhodamine phalloidin. The experiment was performed three times with similar results.

The intoxication with PA-transported fusion toxins is not specific for cancer cells. It depends on the presence of one of the anthrax receptors, which are widely distributed in cells. To enhance specificity towards cancer cells, we used a PA double mutant (mPA) devoid of binding to the natural receptors. MPA was fused to either EGF or to ZHER2, an affibody directed against HER2^24^. As a cellular model system, we used two well characterized esophageal carcinoma cell lines OE21 and OE33. In a previous study, it was shown that the squamous carcinoma cell line OE21 expresses high amounts of EGF receptors on their surface but very little HER2. The adenocarcinoma cell line OE33 on the other hand over-expresses HER2 and a small amount of EGFR (Fig. 3a,b 27). Encompassing these properties, the two cell lines are a useful model system to study the specificity of the re-directed toxin transporters. We intended to analyze the specific uptake of LFN-C3 via the re-directed toxin transporters into either cell line, OE21 or OE33 cells. Therefore, cells were incubated with PA, mPA-EGF or mPA-ZHER2, respectively together with LFN-C3 or left untreated for 4 or 24 (Fig. S1) hours as indicated. As shown in Fig. 4a, OE21 cells treated with mPA-ZHER2 plus LFN-C3 did not round up within 4 and 24 h (Fig. S2), whereas only cells treated with mPA-EGF plus LFN-C3 or PA plus LFN-C3 rounded up within 4 h. In contrast, OE33 cells treated with analogous protein combinations rounded up when incubated with PA plus LFN-C3, mPA-ZHER2 plus LFN-C3 and with mPA-EGF plus LFN-C3 (Fig. 4b). The data show that mPA-ZHER2 mediates entry of LFN-C3 into HER2-positive OE33 cells whereas OE21 cells show no morphological response. Also actin staining of the cells following treatment with the above described protein combinations showed the specificity of mPA-ZHER2 for OE33 cells (Fig. S3). Only in OE21 cells treated with PA plus LFN-C3 or mPA-EGF plus LFN-C3 the actin cytoskeleton clustered, indicating that the fusion-toxin entered the cells. Actin clustering can be visualized better at earlier time points of intoxication, shown in Fig. 5. The pore forming unit mPA-ZHER2 cannot bind sufficiently to OE21 cells and therefore is not able to mediate entry of LFN-C3.

### Quantitative evaluation of the specificity

**Figure 6:**
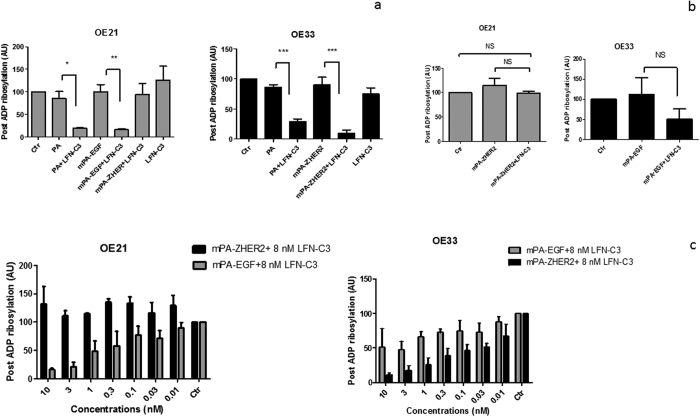
Post-ADP-ribosylation of cell-lysates (**a,b**): OE21 cells and OE33 cells were treated with PA (10 nM), mPA-EGF (10 nM), mPA-ZHER2 (10 nM), LFN-C3 (8 nM) for 4 h, as indicated, washed and lysed. Lysates were than incubated with LFN-C3 in the presence of radio-labeled NAD^+^. Proteins of the lysates were separated by SDS-PAGE. Following drying of the gels labeled protein bands were detected by phosphorimaging and quantified. Labeled actin of an untreated control was set as 100%. Data give the median of three experiments plus standard deviation. Significance was analyzed using GraphPad Prism 5 (***p < 0,001, **p < 0,01, *p < 0,05). Post-ADP-ribosylation of cell-lysates following intoxication as dose response analysis (**c**): OE21 or OE33 cells were treated with increasing concentrations of mPA-EGF (gray, 0.01 nM to 10 nM) or mPA-ZHER2 (black, 0.01 nM to 10 nM) and a fixed concentration of LFN-C3 (8 nM) for 4 h, as indicated. Lysates were prepared and treated as in (**a**). For each experiment a representative Western blot is shown in Fig. S4.

To evaluate the specificity of mPA-ZHER2 in a quantitative manner, we performed a dose response analysis using post-ADP-ribosylation, apoptosis (caspase activity) and cell viability assays. For post-ADP-ribosylation, OE21 and OE33 cells were treated with the proteins indicated. Cells were lysed and the lysates were incubated with LFN-C3 in the presence of radioactive NAD^+^. In this experiment only actin, which was not modified in the living cells by the previous treatment, is labeled. Strong labeling in the lysates thus indicates less modification of actin within the cell. The results shown in Fig. 6 are in line with the morphological studies: In OE21 cells only mPA-EGF guided LFN-C3 into the cytosol, whereas in OE33 both mPA-EGF and mPA-ZHER2 allowed entry of the fusion-toxin into cells. However, mPA-ZHER2 seemed to be more effective. There was no effect of the transporters alone on the cell lines (Fig. 6b). To get more insight into the quantitative differences of the binding/transport capacity of the PA proteins, dose response analysis using different concentrations of the transporters and a fixed concentration of LFN-C3 for intoxication of the two OE cell lines was performed. Figure 6c shows that in OE21 cells the amount of actin ADP-ribosylated through action of the targeted toxin increased with increasing concentrations of mPA-EGF and LFN-C3, whereas there was no ADP-ribosylation detectable in lysates of OE21 cells treated with the highest concentration of mPA-ZHER2 and LFN-C3. Representative Western blots are shown in Fig. S4. In OE33 cells both transporters led to concentration-dependent ADP-ribosylation of actin. However, mPA-ZHER2 was more effective. The calculated effective concentration to get 50% of the maximal effect (EC_50_) of these experiments are depicted in Table 1.

### Effect on cancer cell survival/cell death

**Figure 7:**
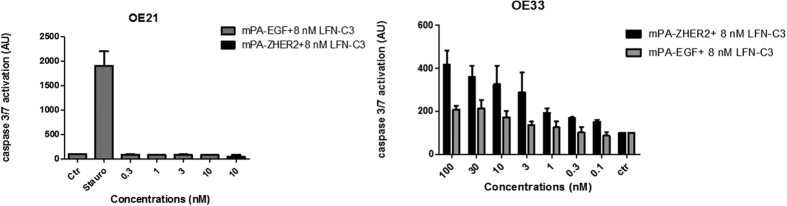
Caspase activity of toxin-treated cells: OE21 or OE33 cells were treated with increasing concentrations of mPA-EGF (gray, 0.1 nM to 100 nM) or mPA-ZHER2 (black, 0.1 nM to 100 nM) and a fixed concentration of LFN-C3 (8 nM) for 24 h, as indicated. Lysates were prepared and tested for caspase 3/7 activity. Staurosporine was used as positive control.

A second approach to study toxin-mediated effects was the detection of tumor cell death. First, we studied caspase activity induced by actin clustering using an Apo1 assay (see materials and methods). OE33 cells responded to toxin treatment with caspase activation and apoptosis in a concentration dependent manner (Fig. 7). In contrast, the highest concentration of the PA protein without addition of LFN-C3 did not induce caspase activation (Fig. S5). In line with the post-ADP-ribosylation, the mPA-ZHER2 transporter was more effective (Fig. 7, EC_50_ shown in Table 1). Surprisingly, OE21 cells did not show detectable caspase activity following intoxication with mPA-EGF or mPA-ZHER2 (Fig. 7). However, poly (ADP Ribose) polymerase (PARP) -cleavage was induced in OE21 cells treated with PA and LFN-C3 and in cells treated with mPA-EGF and LFN-C3 (Fig. S6). Morphological changes, actin clustering and post-ADP-ribosylation proved uptake and action of the catalytic LFN-C3.

**Figure 8:**
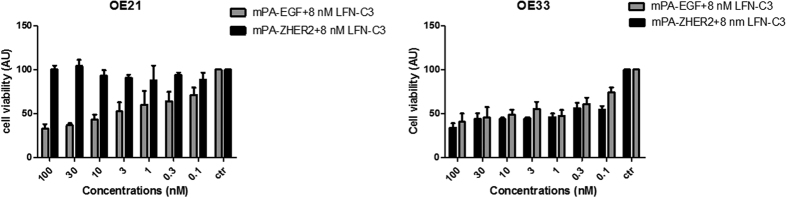
Cell viability of esophageal cancer cells: OE21 or OE33 cells were treated with increasing concentrations of mPA-EGF (gray, 0.1 nM to 100 nM) or mPA-ZHER2 (black, 0.1 nM to 100 nM) and a fixed concentration of LFN-C3 (8 nM), as indicated. Cell viability of the OE21 cells was tested after 24 h with the CellTiter-Blue kit (Promega). Cell viability of the OE33 cells was tested after 48 h with the CellTiter-Glo kit (Promega).

Several forms of cell death are known (e.g. apoptosis, pyroptosis, methuosis) some of which are characterized by missing caspase activity. Therefore, we additionally studied cell viability following intoxication with increasing concentrations of PA proteins and a constant concentration of LFN-C3. In line with the post-ADP-ribosylation, the mPA-ZHER2 transporter together with LFN-C3 did not lead to cell death of OE21 cells. The single components did not significantly change viability of the cells (Fig. S7). Moreover, mPA-EGF combined with LFN-C3 decreased cell-viability of OE21 cells in a concentration-dependent manner (Fig. 8, calculated EC_50_ shown in Table 1). In line with the previous experiments, mPA-ZHER2 leads to slightly more efficient transport of LFN-C3 into OE33 cells compared to mPA-EGF (EC_50_ in Table 1), which was not as obvious in this experiment as compared to former ones.

Comparison of the EC_50_ values of all experiments showed an over 100-fold increase in efficacy of mPA-ZHER2-mediated toxin transport into OE33 cells, which express a higher amount of HER2 compared to OE21 cells (Table 1). In the concentration range tested, there was no measurable toxic effect of mPA-ZHER2 combined with LFN-C3 in OE21 cells, which do weakly express HER2. The data show that OE21 and OE33 cells are a useful test system to evaluate selectivity of redirected toxin transporters. Moreover, LFN-C3 is a useful toxic component with the ability to kill cancer cells with little or no influence on cells which do not express or only weakly present the respective surface receptor of the carrier.

## Discussion

In recent years, much effort has been spent on the identification of surface molecules, exclusively expressed on tumor cells. Although no specific tumor marker has been uncovered so far, some proteins are found enriched on specific cancer cell type surfaces allowing more efficient uptake of cytostatic drugs and immunotoxins. Using two well characterized esophageal cancer cell lines, OE21 as a typical esophageal squamous cell carcinoma (ESCC) showing high expression of EGFR and OE33 esophageal adenocarcinoma (EAC), characterized by high expression of HER2, we established a model system to study the specificity of engineered toxins directed to surface molecules, which are highly expressed on several tumor cells. For example, HER2 is overexpressed in about 20% of breast cancers (Nitta, 2016).

Most bacterial toxins are composed of several, independent domains, which encompass diverse functions. Targeting a bacterial toxin to a specific surface molecule can thus be achieved by exchanging the receptor binding domain of the toxin with a receptor binding domain of another protein having high affinity towards the chosen receptor^32^. Following endocytosis of the chimeric toxin the toxin’s catalytic domain has to be released from the endosome into the cytosol. This translocation step is not well understood. It requires hydrophobic amino acid stretches used for insertion into the endosomal membrane. This hydrophobic domain may limit the successful expression of the recombinant protein and therefore the functionality of several engineered toxins.

Instead of generating a fusion toxin, we used a targeted toxin transporter based on the anthrax toxin^33^. Seven monomers of this transporter bind to the cell surface and form a pore in the endosomal membrane. Binding of the monomers to the natural anthrax receptors is prohibited by two mutations. Instead, the mutated monomers are re-directed to EGFR or HER2, receptors overexpressed on the surface of cancer cells, by fusion of EGF or of an affibody directed against HER2 to the N-terminus of mPA, respectively^24^. The first advantage of this dimeric system is the higher avidity of the transporter towards the cancer cell, because each subunit of the heptameric pore is fused to EGF or ZHER2 and therefore has seven possible binding sites on the cell surface. The second advantage is that no translocation domain of the catalytic part is necessary for its transport into the cytosol, which often hampers recombinant expression. Using an established targeted transporter system many different enzymes can potentially be studied. Here, we used the *Photorhabdus luminescent* ADP-ribosyltransferase C3. This toxin modifies actin at a unique site (threonine-148) resulting in actin aggregation by inhibiting binding of the actin sequestering protein thymosin-β4 and leading to cell death^34^.

The two cell lines of the two main histotypes of esophageal cancer expressing divergent amounts of the two targeted receptors, EGF-receptor (EGFR) and HER2 emerge as an ideal model system to compare surface receptor-dependent toxin uptake and efficiency. Although both cell lines express EGFR and HER2, the stronger HER2 expression of OE33 mediate an about 100-fold higher specificity towards mPA-ZHER2 mediated intoxication. OE21 cells marginally express HER2, however, there is only low (if at all) intoxication of the cells with mPA-ZHER2 used as toxin transporter. This indicates that a crucial number of surface molecules must be accessible for successful binding and/or uptake of the toxin ensuring low unspecific toxicity. In former studies, Mechaly *et al*. used mPA-EGF for delivery of a fusion of LFN and the catalytic domain of diphtheria toxin into human epidermoid carcinoma cells (A431) which have a very strong expression of EGFR. Specificity over EGFR negative Chinese Hamster Ovary (CHO) cells was at least 1:1000 24. Here, we used two mammalian cell-lines (OE21 and OE33) expressing the EGF and HER2 receptors in different amounts and still reached a selectivity of about 1:100, respectively. Besides the total amount of surface standing receptors, other cellular properties like membrane fluidity (for insertion of the toxin pore) and chaperone content (for refolding the transported catalytic toxin part) may be crucial for the activity of the targeted toxin. Our model system excludes such differences largely, because of the two cell lines acting crossover as positive and negative control, respectively. Compared to other studies using PA as a protein transport system (for excellent review see ref. 35), we used a different catalytic toxin, which targets the cytoskeleton instead of blocking protein synthesis. The *Photorhabdus luminescence* toxin TccC3 catalyzes the ADP-ribosylation of actin thereby forcing uncontrolled actin polymerization and subsequent cell death^29^. The modification occurs with monomeric and polymerized actin and should be completely independent from the cell cycle and state of cells, which still has to be shown^26^. Moreover, blocking the functionality of the actin cytoskeleton should inhibit migration of tumor cells, which is a prerequisite for metastasis. In OE33 cells toxin-induced actin aggregation induces apoptosis. In OE21 cells TccC3 also induces cell death and PARP-cleavage. Therefore, the two cell lines used in this study build a useful model system to analyze several toxins as potential protein cargoes delivered by the targeted toxin transporters mPA-EGF and mPA-ZHER2.

## Methods

### Construction of the LFN-fusion toxins

The N-terminal 263 amino residues of LF (LFN) were amplified and cloned into the pET-28a (+) vector (Novagen, EMD Biosciences). PCR was performed with following primers: sense primer 5′-cat atg gcg ggc ggt cat ggt ga-3′ and antisense primer 5′-gga tcc ccg ttg atc ttt aag ttc ttc caa gg-3′. For amplification of the ADP-ribosyltransferase domain hvr of TccC3 (residues 679–960), PCR was performed with sense primer 5′-gga tcc atg cca aca att gca gaa cgc ata gca-3′ and antisense primer 5′-ctc gag tta tct ctt atg agg ttt tac att ttt aag c-3′. The PCR product of TccC3hvr with flanking BamHI and XhoI restriction sites was cloned into the LFN-pET-28a (+) vector. Each construct was verified by sequencing. For construction of the catalytically inactive mutant, LFN-C3(E943A) was generated by quick-change PCR, using the following primers: sense 5′ gga ccg gta aat gat gca gca att tca ttt ctg aca ac 3′ and antisense primer 5′-gtt gtc aga aat gaa att gct gca tca ttt acc ggt cc-3′. The construct was verified by sequencing. The PA Φclamp mutant (PA(F427A)) was generated as described in ref. 31.

### Cell culture/Intoxication

OE21 and OE33 cells were grown in RPMI 1640 medium supplemented with 10% fetal calf serum, L-Glutamine (2 mM), streptomycin (4 mM) penicillin (4 mM) and 1% non-essential amino acids. HeLa cells were cultivated in Dulbecco’s Modified Eagle medium supplemented with 10% fetal calf serum, L-Glutamine (2 mM), streptomycin (4 mM) penicillin (4mM) and 1% non-essential amino acids. All cell lines were cultivated in humidified atmosphere of 5% CO_2_ at 37 °C and checked negative for mycoplasma monthly.

For intoxication, OE21 and OE33 cells were treated in culture medium (0.5% FCS) with the concentrations and times indicated and a confluency between 60 to 90%. Cells were then washed once with phosphate-buffered saline (PBS) and lysed with 50 mM Tris-HCl pH 7.4, 100 mM NaCl, 2 mM MgCl_2_, 10% glycerol and 1% Nonidet P-40 for 10 min at 4 °C. Cells were harvested using a rubber policeman and centrifuged at 14000 RPM for 15 min at 4 °C.

Protein concentration was measured and adjusted using the bicinchoninic acid assay or the Roti^®^-quant following manufacturer’s instructions.

### Actin staining

Cells were seeded on glass coverslips overnight, then intoxicated in culture medium (0.5% FCS) for 90 min or 4 h with according concentrations. After washing with PBS, cells were fixed in fixation buffer (3.7% paraformaldehyde, 0.1% Triton in PBS) for 10 min at room temperature. Cover slips were washed three times with PBS and incubated for 1 h with Phalloidin-TRITC (Tetramethyl rhodamine B isothiocyanate). Coverslips were washed 3 times then dried overnight with Moviol supplemented with DAPI (4′,6-diamidino-2-phenylindole) for nuclear staining. Staining was visualized by fluorescence microscopy.

### *In vitro* ADP-ribosylation experiment

For ADP-ribosylation of actin and RhoA, cell lysates were incubated in a reaction mix (25 nM TEA pH 7.5, 10 mM DTT, 15 mM MgCl_2_ and 12 mM thymidine) with 5 μg/mL of toxins and 0.5 μCi of radioactive [^32^P] NAD. The reaction was incubated for 15 min at 37 °C. Proteins were separated by SDS-PAGE (12.5%) and gels were dried for 1h followed by autoradiography. For loading control, RhoA was additionally modified by adding ADP-ribosylating toxin C3 (*Clostridium botulinum*) to the reaction mix.

### Purification of proteins

BL21 (DE3) bacteria were transformed with 100 ng DNA. Protein expression was induced with 75 μM isopropyl β-D-1-thiogalactopyranoside (IPTG) then cells were grown at 28 °C overnight in Luria-Bertani (LB) medium supplemented with antibiotics (Ampicillin or Kanamycin).

Bacteria were harvested by centrifugation at 6000 rpm for 15 min at 4 °C and lysed in lysis buffer (300 mM NaCl, 20 mM Tris-HCl, 500 μM ethylenediaminetetraacetic acid (EDTA) pH 8.5) supplemented with 5 μg/ml DNAse, 1 mg/ml Lysozym and 200 mM phenylmethanesulfonyl fluoride (PMSF). Cells were sonicated and the lysates centrifuged for 45 min at 14000 rpm at 4 °C. The supernatant was incubated with Protino-Ni-IDA (Macherey-Nagel) for 2 h at 4 °C and loaded on PD-10 columns. His-tagged proteins were eluted with an elution buffer (500 mM NaCl, 20 mM Tris-HCl, 0.05% Tween 20, 1 mM EDTA, 500 mM Imidazole pH 8.5). After elution, proteins were dialyzed overnight in 150 mM NaCl, 50 mM Tris-HCl, and 1 mM EDTA pH 8.5. Proteins were then centrifuged at 14000 rpm for 15 min and stored in glycerol (40%). Purity of the recombinant proteins was about 80% (LFN-C3) and about 20% (mPA-EGF, mPA-ZHER2) of total protein.

### Caspase 3/7 activation measurements, Cell viability measurements

Cells were seeded in a 96 wells plate overnight. Cells were then intoxicated in culture medium (0.5% FCS) with indicated concentrations for 24 h. Staurosporine was added for 17 h. Caspase 3/7 activation was measured using the *Apo-ONE*^®^ Homogenous Caspase-3/7 Assay (Promega), following the manufacturer’s protocol. Cells were incubated with reagent for 1 h at room temperature. Cell viability of OE21 cells was measured using the CellTiter-Blue^®^ Cell Viability kit (Promega), following the manufacturer’s protocol. Cells were incubated with reagent for 1 h at 37 °C. Because no cell death could be measured using the CellTiter-Blue^®^ Cell Viability kit, viability of OE33 cells was measured using the CellTiter-Glo*^®^ Kit* (Promega), with higher sensitivity, following the manufacturer’s protocol. Cells were incubated at RT for 10 min.

### Indirect immunofluorescence

Cells were grown on coverslips, fixed in 2% PFA, washed in PBS and permeabilized in 0.5% (v/v) Tritron X-100 in PBS. After PBS washing, cells were incubated with blocking buffer (PBS containing 5.0% (v/v) normal goat serum and 0.3% (v/v) Tritron X-100). Diluted primary antibodies (mouse anti-human EGFR clone H11 (Dako) and polyclonal rabbit anti-human c-erbB-2 Oncoprotein (Dako)) were incubated overnight at 4 °C. Cells were rinsed with PBS and 1:200 diluted fluorescently labelled secondary antibodies (goat-anti-mouse IgG-Alexa488 (Invitrogen) and goat-anti-rabbit IgG-Alexa568 (Invitrogen)), were incubated for 1 hour at RT. After washing with PBS and distilled water, cell nuclei were counterstained with DAPI (Vector Laboratories). Stainings were evaluated at a fluorescence microscope (Axioplan2 imaging microscope equipped with a Plan-Neofluar 40x/1.3 oil objective, Carl Zeiss MicroImaging) with slider module. Image stacks at 0.9 μm intervals were taken and converted into 3D view by AxioVision software (Carl Zeiss MicroImaging).

### Western Blot

Preparation of total protein was perfomed using the Qproteome™ Mammalian Protein Prep Kit (Qiagen) according to the manufacturer’s protocols. Determination of protein concentration was performed using the DC Protein Assay (Bio-Rad) according to the manufacturer’s protocols. 15 μg of total protein extracts per lane were loaded onto 8% polyacrylamide gels. Proteins were transferred onto Amersham™ Hybond™ ECL Nitrocellulose Membrane (GE Healthcare) by Semi-Dry blot. The membrane was blocked in 5% (m/v) nonfat dried milk powder in Tris buffered saline with Tween (TBST). Then the primary antibodies diluted in 5% (m/v) nonfat dried milk powder in TBST (mouse anti-human EGFR clone H11 (Dako), mouse anti-β-Actin clone AC-15 (Sigma-Aldrich)) or 5% BSA in TBST (polyclonal rabbit anti-HER2/ErbB2 (Cell Signaling)) were incubated. After HRP conjugated secondary antibody (Dianova) incubation, the membrane was incubated with ECL reagents (Thermo Scientific). Chemiluminescence detection and imaging was done with the Fusion Fx7 System and the FusionCapt Advance Software (Vilber Lourmat).

### Statistics

Statistical significance of the data was analyzed by one-way-Anova, including all data measured and by performing a Bonferroni test for selected pairs of columns as indicated in the figures. All data were normalized to the untreated control.

For the EC50 calculation: The experiments were plotted using Graphpad 5 and the concentrations were transformed to a logarithm. The non-linear regression fit was chosen and the dose response stimulation was defined as log agonist vs normalized response (to the untreated control).

## Additional Information

**How to cite this article**: Zahaf, N.-I. *et al*. Targeted delivery of an ADP-ribosylating bacterial toxin into cancer cells. *Sci. Rep.* **7**, 41252; doi: 10.1038/srep41252 (2017).

**Publisher's note:** Springer Nature remains neutral with regard to jurisdictional claims in published maps and institutional affiliations.

## Supplementary Material

Supplementary Information

## Figures and Tables

**Table 1:** EC_50_ values of targeted toxins.

EC_50_ Values				
	OE21		OE33	
Assay	mPA-ZHER2	mPA-EGF	mPA-ZHER2	mPA-EGF
ADP-ribosylation	>10	0.5	0.1	6
Caspase 3/7 activation	>10	>10	2.5	5.2
Cell viability	>100	5	2	7
