# Supplementary material for: Targeted delivery of an ADP-ribosylating bacterial toxin into cancer cells
Source: Sci Rep. 2017 Jan 27;7:41252. doi: 10.1038/srep41252 (PMC5269596; doi:10.1038/srep41252)
Supplement: Supplementary Information [file srep41252-s1.pdf]

Supplements to

Targeted delivery of an ADP-ribosylating bacterial toxin into cancer cells

Zahaf N.-I.[1*], Lang A.E.[1*], Kaiser L[1]., Fichter C.D.[2,3] , Lassmann S.[2,3,4,5,6], McCluskey A.[7], Augspach, A.[1], Aktories, K.[1,3,5] and Schmidt G.[1,3,#]

Fig. S1: Cell rounding is induced by PA plus LFN-C3 but not by PA plus LFN-C3(E943A). (PA(F427A), Φclamp) combined with LFN-C3 does not induce cell rounding. OE21 cells were incubated with the following proteins as indicated: PA (10 nM), mPA-EGF (10nM), PA(F427A) (10 nM), LFN-C3 (8 nM), LFN-C3(E943A) (8nM). Photos were taken after 24 h of incubation. A typical result of 2 independent experiments is shown.

Fig. S2: Cell specific cell rounding induced by LFN-C3 mediated by different PA transporters: OE21 and OE33 cells were incubated with the following proteins as indicated: PA (10 nM), mPA-EGF (10 nM), mPA-ZHER2 (10 nM), LFN-C3 (8 nM). Photos were taken after 24 h of incubation. A typical result of 3 independent experiments is shown.

Fig. S3: Uncontrolled actin clustering in esophageal cancer cells induced by LFN-C3 mediated by different PA transporters: OE21 (a) cells and OE33 (b) cells were treated with PA (10 nM), mPA-EGF (10 nM), mPA-ZHER2 (10 nM), LFN-C3 (8 nM) as indicated. After 4 h cells were fixed and stained with Rhodamine phalloidin. The experiment was performed three times with similar results.

Fig. S4: Representative Western blots for each experiment of Fig.6: Post-ADP-ribosylation of cell-lysates (a, b): OE21 cells and OE33 cells were treated with PA (10 nM), mPA-EGF (10 nM), mPA-ZHER2 (10 nM), LFN-C3 (8 nM) for 4 h as indicated, washed and lysed. Lysates were than incubated with LFN-C3 in the presence of radio-labeled NAD^+. Proteins of the lysates were separated by SDS-PAGE. Following drying of the gels labeled protein bands were detected by phosphorimaging and quantified. Labeled actin of an untreated control was set as 100%. Data give the median of three experiments plus standard deviation. Significance was analyzed

using GraphPad Prism 5 (*** : p < 0,001, ** : p < 0,01, * : p < 0,05). Post-ADP-ribosylation of cell-lysates following intoxication as dose response analysis (c): OE21 or OE33 cells were treated with increasing concentrations of mPA-EGF (gray, 0.01 nM to 10 nM) or mPA-ZHER2 (black, 0.01 nM to 10 nM) and a fixed concentration of LFN-C3 (8 nM) for 4 h, as indicated. Lysates were prepared and treated as in a.

Fig. S5: Caspase activity of toxin-treated cells: OE33 cells were treated with PA (10 nM), mPA-EGF (10 nM) or mPA-ZHER2 (10 nM), LFN-C3 (8 nM) or Staurosporine (10 µM) for 24 h, as indicated. Lysates were prepared and tested for caspase 3/7 activity.

Fig. S6: PARP-cleavage detected in OE21 and OE33 cells: Fig. S4: PARP cleavage detected in OE21 and OE33 cells: OE21 and OE33 cells were treated with PA (10 nM), mPA-EGF (10 nM), mPA-ZHER2 (10 nM), LFN-C3 (8 nM) for 24h as indicated. PARP cleavage was then detected by Western blot. The untreated control was set to 1 and PARP cleavage represents X fold of control. The Data give the median of three experiments plus standard deviation. Significance was (*** : p < 0,001, ** : p < 0,01, * : p < 0,05).

Fig. S7: Cell viability of OE21 cells: OE21 cells were treated with PA (10 nM), mPA-EGF (10 nM) or mPA-ZHER2 (10 nM), LFN-C3 (8 nM) or Staurosporine (10 µM) for 24 h, as indicated. Cell viability was measured with CellTiter-Blue kit (Promega).

Fig S1

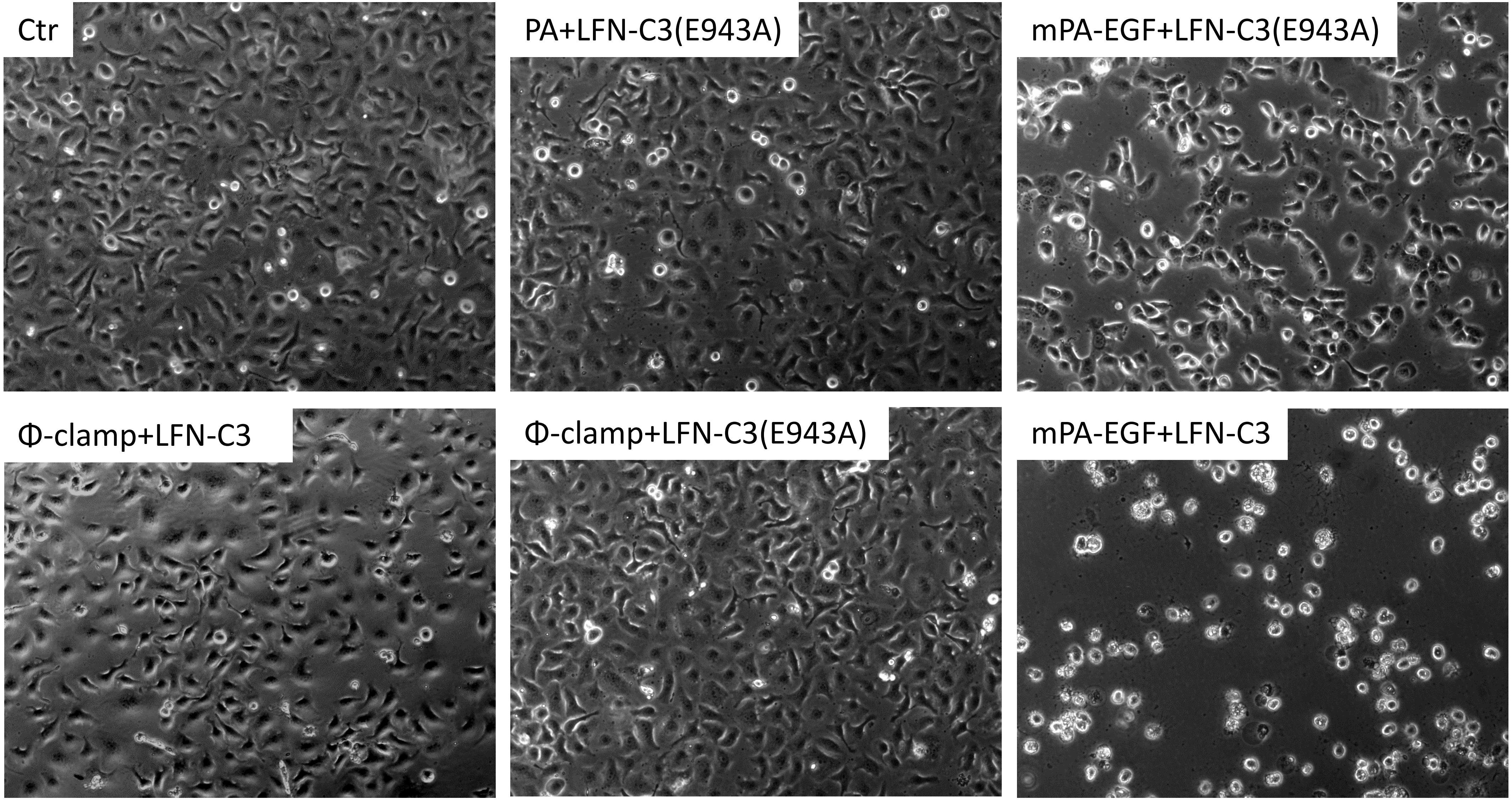
Ctr
PA+LFN-C3(E943A)
mPA-EGF+LFN-C3(E943A)
Φ-clamp+LFN-C3
Φ-clamp+LFN-C3(E943A)
mPA-EGF+LFN-C3

Fig S2

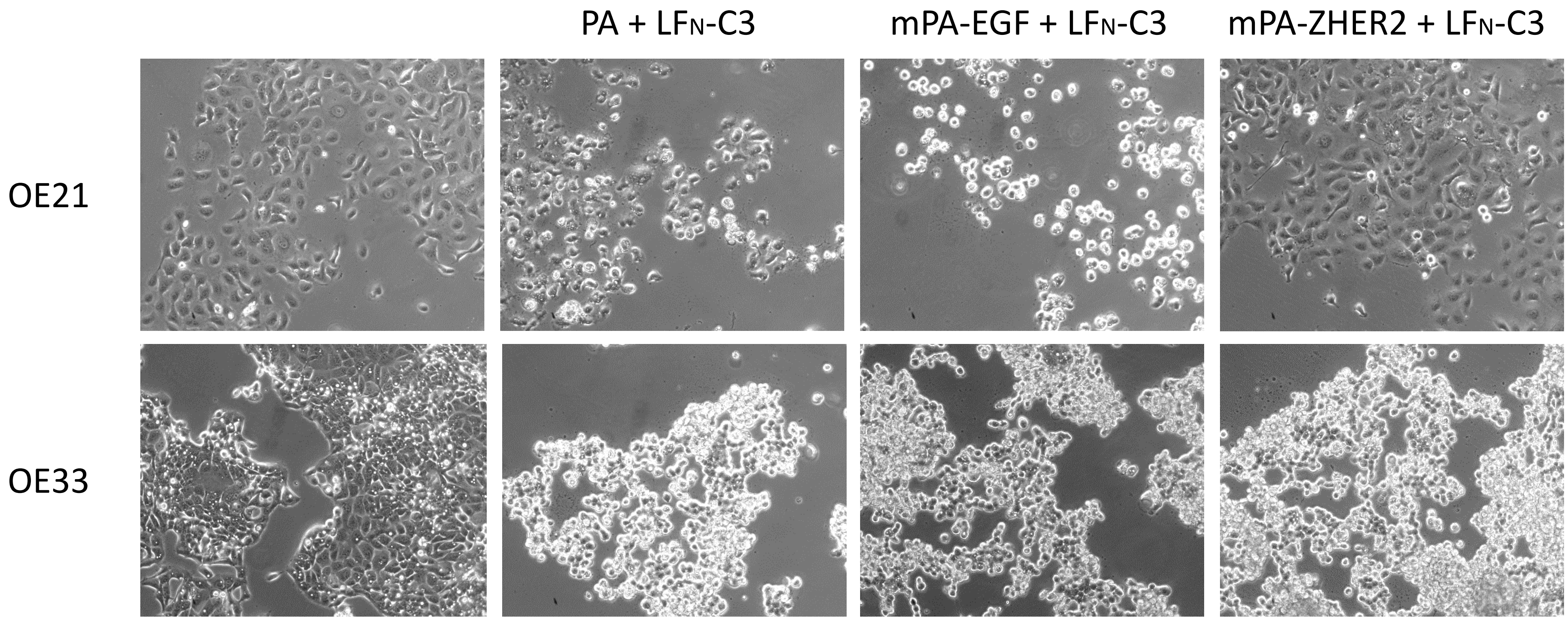

Fig.S3

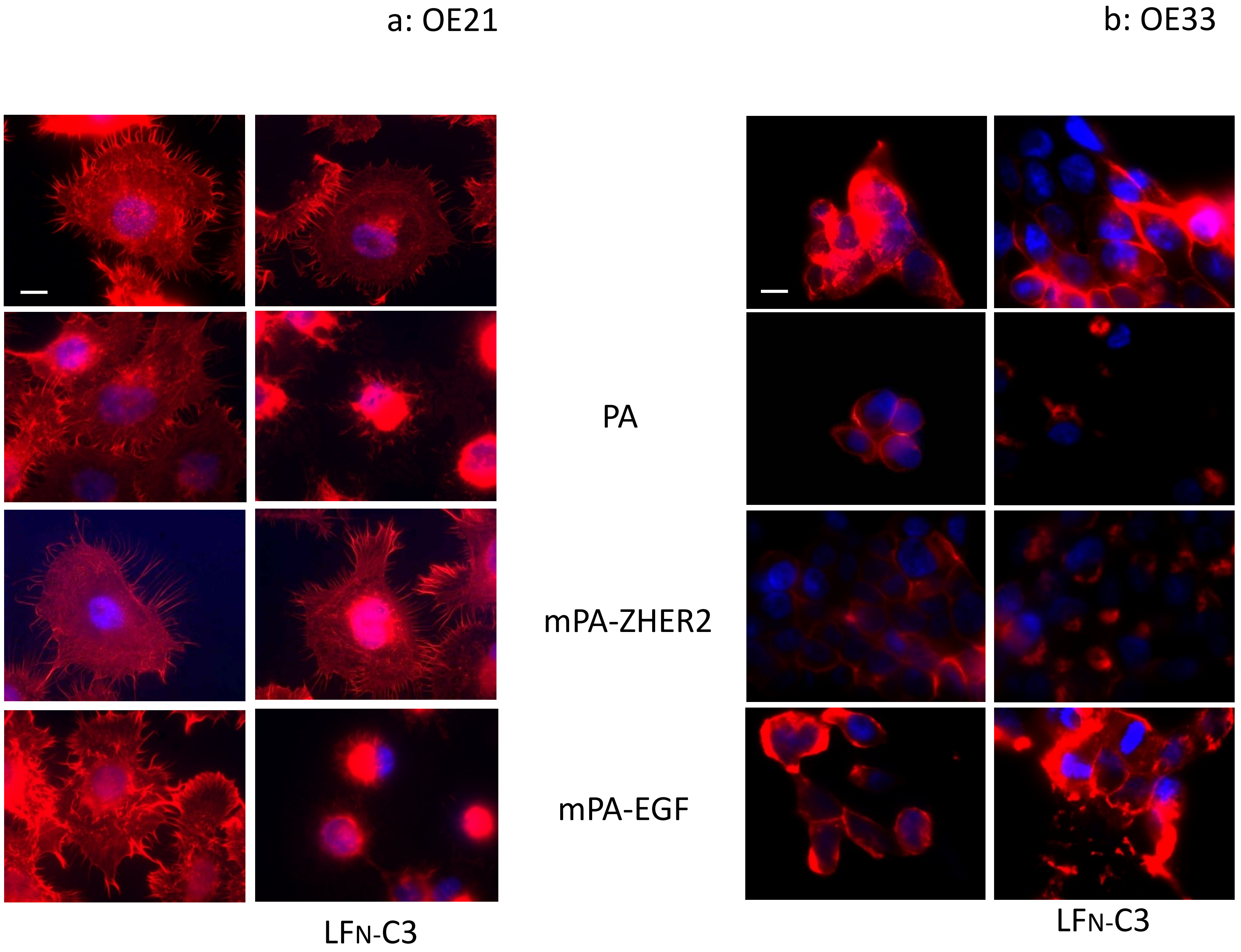

Fig S4

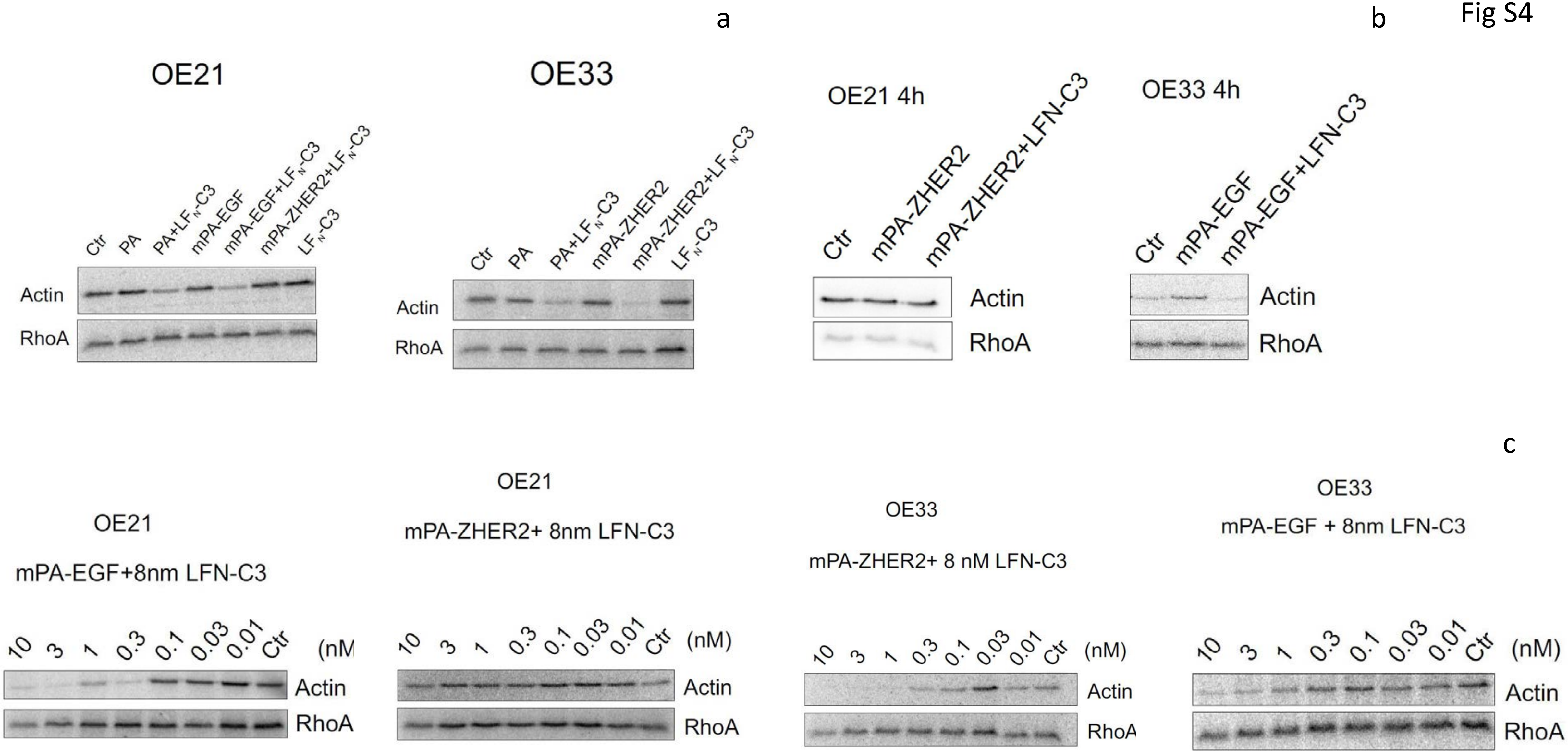
a
b
OE21
Ctr
PA
PA+LF N-C3
mPA-EGF
mPA-EGF+LF N-C3
mPA-ZHER2+LF N-C3
LF N-C3
Actin
RhoA
OE33
Ctr
PA
PA+LF N-C3
mPA-ZHER2
mPA-ZHER2+LF N-C3
LF N-C3
Actin
RhoA
OE21 4h
Ctr
mPA-ZHER2
mPA-ZHER2+LFN-C3
Actin
RhoA
OE33 4h
Ctr
mPA-EGF
mPA-EGF+LFN-C3
Actin
RhoA
c
OE21
mPA-EGF+8nm LFN-C3
10 3 1 0.3 0.1 0.03 0.01 Ctr (nM
Actin
RhoA
OE21
mPA-ZHER2+ 8nm LFN-C3
10 3 1 0.3 0.1 0.03 0.01 Ctr (nM)
Actin
RhoA
OE33
mPA-ZHER2+ 8 nM LFN-C3
10 3 1 0.3 0.1 0.03 0.01 Ctr (nM)
Actin
RhoA
OE33
mPA-EGF + 8nm LFN-C3
10 3 1 0.3 0.1 0.03 0.01 Ctr (nM)
Actin
RhoA

Fig. S5

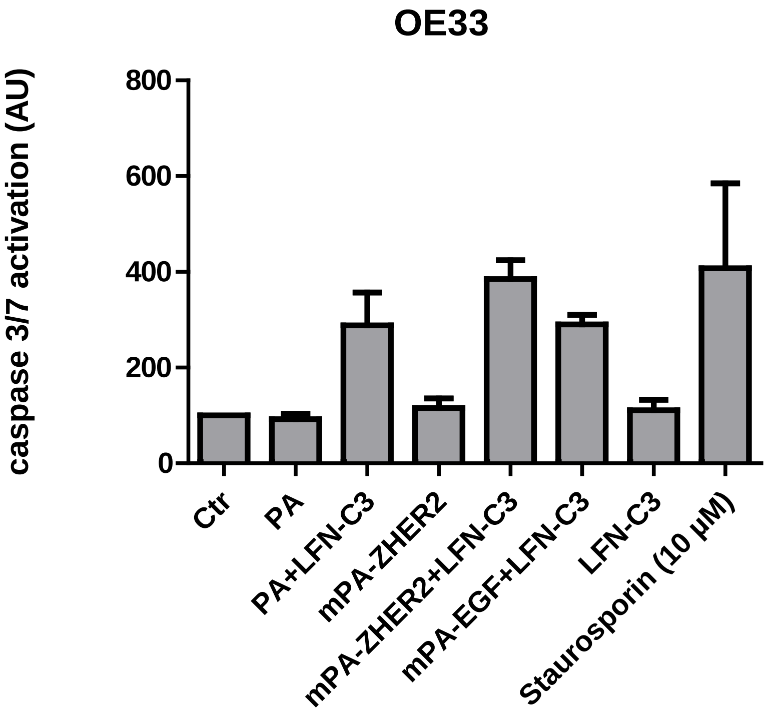
OE33
caspase 3/7 activation (AU)
800
600
400
200
0
Ctr
PA
PA+LFN-C3
mPA-ZHER2
mPA-ZHER2+LFN-C3
mPA-EGF+LFN-C3
LFN-C3
Staurosporin (10 µM)

Fig. S6

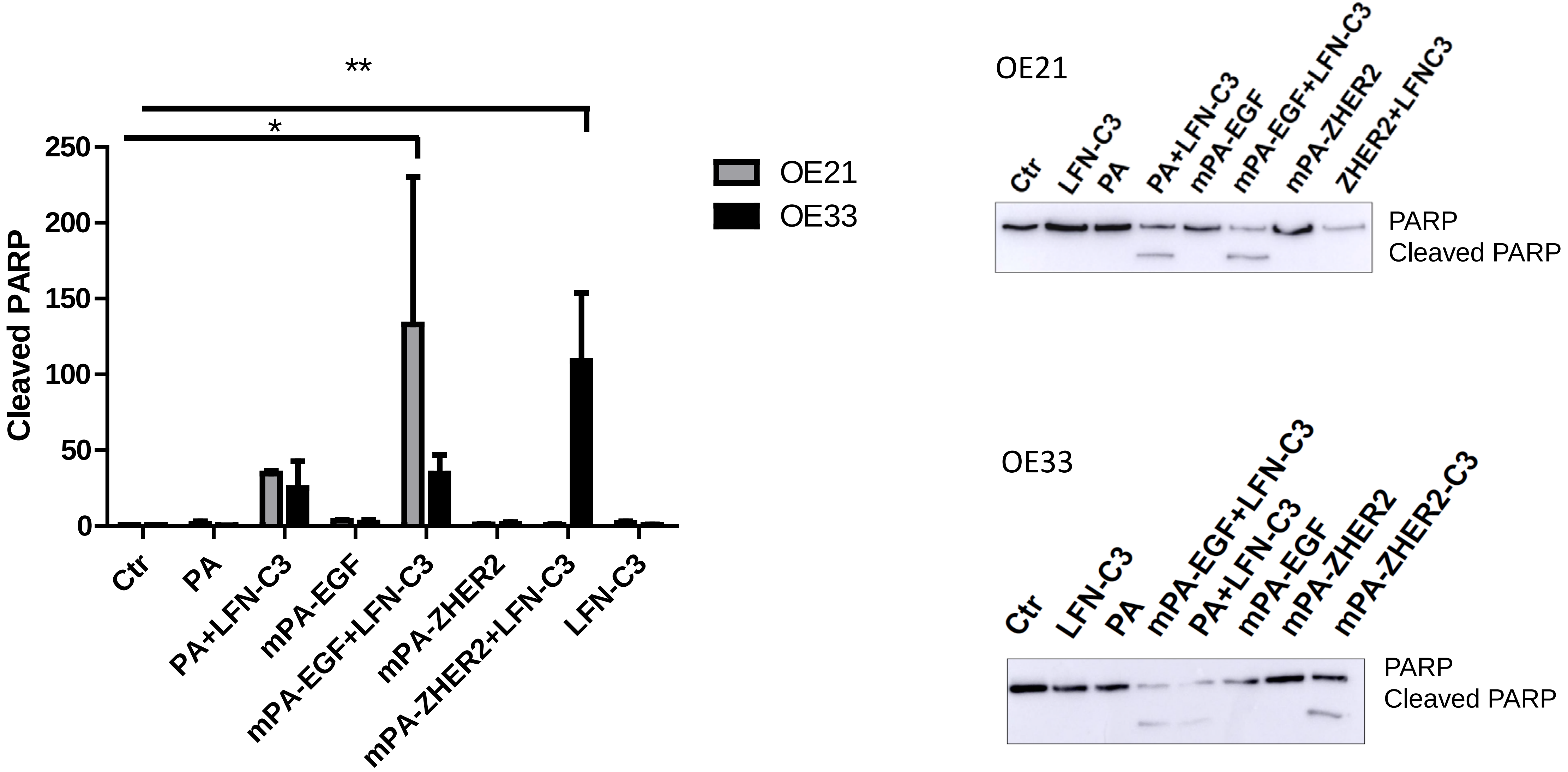

**
*
Cleaved PARP
250
200
150
100
50
0
Ctr
PA
PA+LFN-C3
mPA-EGF
mPA-EGF+LFN-C3
mPA-ZHER2
mPA-ZHER2+LFN-C3
LFN-C3
OE21
OE33
OE21
Ctr
LFN-C3
PA
PA+LFN-C3
mPA-EGF
mPA-EGF+LFN-C3
mPA-ZHER2
ZHER2+LFNC3
PARP
Cleaved PARP
OE33
Ctr
LFN-C3
PA
mPA-EGF+LFN-C3
PA+LFN-C3
mPA-EGF
mPA-ZHER2
mPA-ZHER2-C3
PARP
Cleaved PARP

Fig. S7

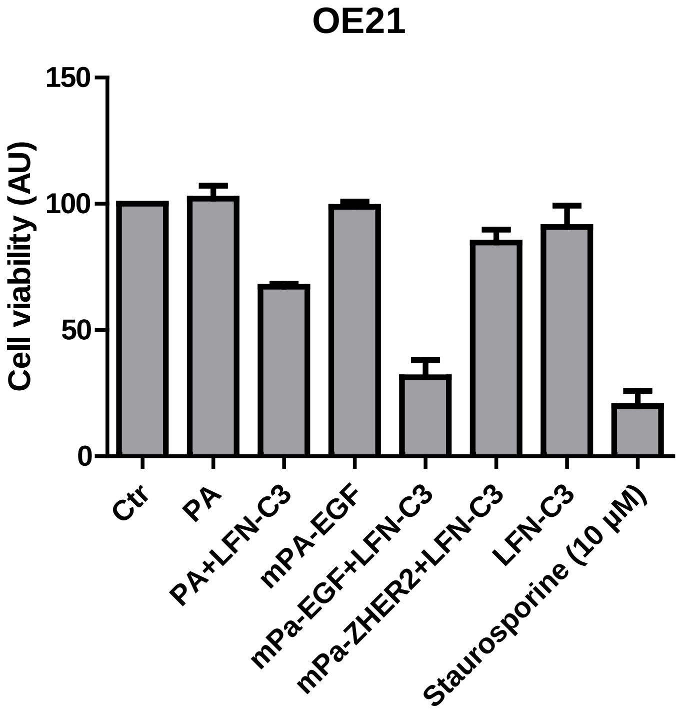

OE21
Cell viability (AU)
150
100
50
0
Ctr
PA
PA+LFN-C3
mPA-EGF
mPa-EGF+LFN-C3
mPa-ZHER2+LFN-C3
LFN-C3
Staurosporine (10 μM)
